# Adventitial adaptive immune cells are associated with ascending aortic dilatation in patients with a bicuspid aortic valve

**DOI:** 10.3389/fcvm.2023.1127685

**Published:** 2023-03-28

**Authors:** Alexander H. J. Staal, Kimberley R. G. Cortenbach, Mark A. J. Gorris, Lieke L. van der Woude, Mangala Srinivas, Robin H. Heijmen, Guillaume S. C. Geuzebroek, Nimrat Grewal, Konnie M. Hebeda, I. Jolanda M. de Vries, Marco C. DeRuiter, Roland R. J. van Kimmenade

**Affiliations:** ^1^Department of Tumor Immunology, Radboud Institute for Molecular Life Sciences, Radboud University Medical Center, Nijmegen, Netherlands; ^2^Division of Immunotherapy, Oncode Institute, Radboud University Medical Center, Nijmegen, Netherlands; ^3^Department of Pathology, Radboud University Medical Center, Nijmegen, Netherlands; ^4^Cell Biology and Immunology, Wageningen University and Research, Wageningen, Netherlands; ^5^Department of Cardiothoracic Surgery, Radboud University Medical Center, Nijmegen, Netherlands; ^6^Department of Cardiothoracic Surgery, Leiden University Medical Center, Leiden, Netherlands; ^7^Department of Anatomy and Embryology, Leiden University Medical Center, Leiden, Netherlands; ^8^Department of Cardiology, Radboud University Medical Center, Nijmegen, Netherlands

**Keywords:** thoracic aorta aneurysm, aortic dissection, multiplex immunohistochemistry, bicuspid aortc valve, inflammation, tertiary lymphoid structures, auto-inflammation

## Abstract

**Background:**

Bicuspid aortic valve (BAV) is associated with ascending aorta aneurysms and dissections. Presently, genetic factors and pathological flow patterns are considered responsible for aneurysm formation in BAV while the exact role of inflammatory processes remains unknown.

**Methods:**

In order to objectify inflammation, we employ a highly sensitive, quantitative immunohistochemistry approach. Whole slides of dissected, dilated and non-dilated ascending aortas from BAV patients were quantitatively analyzed.

**Results:**

Dilated aortas show a 4-fold increase of lymphocytes and a 25-fold increase in B lymphocytes in the adventitia compared to non-dilated aortas. Tertiary lymphoid structures with B cell follicles and helper T cell expansion were identified in dilated and dissected aortas. Dilated aortas were associated with an increase in M1-like macrophages in the aorta media, in contrast the number of M2-like macrophages did not change significantly.

**Conclusion:**

This study finds unexpected large numbers of immune cells in dilating aortas of BAV patients. These findings raise the question whether immune cells in BAV aortopathy are innocent bystanders or contribute to the deterioration of the aortic wall.

## 1. Introduction

Thoracic aortic aneurysm is a prevalent yet lethal condition that is often silent until presentation as an acute aortic syndrome (AAS) (1, 2), such as a dissection or rupture (3). An AAS has a very high mortality rate (4), if not treated surgically in time. Clinical care is focused on stringent blood pressure control and timely preventive aortic replacement surgery (5). The timing of surgery is mainly based on expert consensus and is complicated by an incomplete understanding of aortopathies (6), insufficient pharmacological therapies (7, 8) and most importantly: the poor predictive value of the aortic diameter for AAS development (9).

Bicuspid aortic valve (BAV) is one of the common risk factors for ascending aorta aneurysm and thus AAS (10). A BAV is an aortic valve consisting of two valve leaflets due to congenital non-separation of two of the three aortic valve cusps. BAV is the most common congenital heart defect, affecting 1–2% of the general population (6, 10). The majority of patients with BAV will develop aortic dilatation, an occurrence that increases with age (10). The development of BAV associated aortopathies is not fully understood. Genetics and abnormal flow patterns are implicated, although the precise mechanisms and their value in predicting outcomes remain unclear (6, 11–16). No effective prognostic tools from imaging, biomarkers or genetics have emerged, limiting the possibility to select patients at risk of developing a dissection (10). Moreover, current understanding has not led to medical therapy of BAV aortopathy beyond general anti-hypertensive drugs (clinicaltrails.gov NCT01202721).

Inflammation is seen as an important player in vascular diseases, ranging from hypertension to atherosclerosis and has proven to be an effective therapeutic target (17–20). Strikingly, at first glance BAV aneurysmatic aortas do not demonstrate inflammation (21–24). However, inflammation-associated genes are upregulated in BAV aortopathy (25). To date, potential ongoing inflammatory processes within BAV aneurysms have not been studied at a cellular level with state-of-the-art techniques.

Inflammation is a protective response involving blood vessels, molecular mediators, and many cell types of the immune system. Immune cells can be studied by using markers to specifically identify cell subsets exerting particular functions. The presence of high numbers of B cells in combination with neovascularization can be indicative for chronic inflammation, whereas neutrophils indicate acute inflammation (26, 27). Characterization of T cell subsets, dendritic cell (DC) subsets and macrophage polarization can be used to investigate the extent of involvement and activation status of the immune system (28–30).

In this study we aimed to provide an overview of inflammatory processes in ascending aorta dilatation in patients with BAV. Inflammatory processes were studied with a wide variety of markers, focusing on both the innate and the adaptive immune system. An eight-color immunohistochemistry (IHC) method with automated quantitative analysis was applied to detect differences in immune cell involvement between non-dilated, dilated and dissected aortas. These finding have led to new hypotheses which could explain an additional factor in the pathogenesis of an aortic dissection in BAV patients.

## 2. Materials and methods

### 2.1. Patient samples

Patient material was collected from 3 academic centers in the Netherlands; (1) patients with a BAV undergoing elective surgical repair of ascending aorta aneurysms or acute surgical repair of an acute Stanford type A aortic dissection at the Radboudumc in Nijmegen, (2) patients with a BAV undergoing elective surgical replacement of the aortic valve with or without concomitant proximal aortic replacement at the Leiden University Medical Centre, Leiden, (3) post-mortem non-dilated BAV aortic wall samples from the Heart Valve Bank of the Erasmus Medical Centre in Rotterdam. The Leiden and Rotterdam samples have been used in a prior study (31). Approval by the medical ethics committees of the institutions was obtained before the start of the study conformed to the principles outlined in the Declaration of Helsinki (2017-3196).

The study population was divided in three groups: (1) non-dilated (*n* = 13) defined as a maximum ascending aorta diameter below 40 mm; (2) dilated (*n* = 10) defined as an ascending aorta diameter of 45 mm and larger and (3) hyperacute dissection (*n* = 4) defined as patients with a Stanford type A dissection proven on CT or trans-thoracic ultrasound and symptoms for less than 24 h, in accordance with the IRAD definition (32).

### 2.2. Tissue processing

All dilated and dissection samples were obtained during surgical replacement of the aorta. The dilated samples were taken from the most severely affected (maximal diameter) part of the aorta as assessed by the surgeon. The dissection samples were taken from the area of the entry tear and the immediate distal portion down-stream. In the case of a dissection the intimal flap from the propagating tear and the standing adventitia portions of the aorta were embedded. The thrombotic tissue adhering to the entry and propagating tear were excluded from analysis. All samples were formalin fixed and paraffin embedded: samples were fixed directly after excision in buffered 4% formaldehyde for at least 24 h. Samples were carefully embedded in paraffin to include all aorta layers. Full thickness transverse sections of 4 μm were mounted on silane coated glass slides (New Silane III, MUTO PURE CHEMICALS, Japan).

### 2.3. Multiplex immunohistochemistry

Tissue sections were stained with two multiplex IHC panels (Table 1); one for the detection of lymphocytes and dendritic cells, one for myeloid cells and vascularization. Antibodies and panels were validated and optimized as described in great detail previously by us and others (33–36). The staining procedure consisted of six consecutive tyramide signal amplification stains with an antigen stripping step between all stains. The fluorophore remained on the target after the antigen stripping step resulting in eight simultaneous colors on one slide.

**Table 1:** The two eight-color IHC panels used for identification of cells of the adaptive and the innate immune system.

Markers (clone) per panel			
Adaptive immune cell panel		Innate immune cell panel	
DAPI		DAPI	
CD3 (SP7)		CD68 (PG-M1)	
CD8 (C8/144B)		CD206 (CL0387)	
CD20 (L26)		CD15 (MMA)	
CD1c (2F4)		CD31 (JC70A)	
FoxP3 (236A/E7)		MMP9 (polyclonal)	
CD45RO (UCHL-1)		GM-CSF (polyclonal)	
Cell phenotype per panel			
Autofluorescence (elastin fibers)		Autofluorescence (elastin fibers)	
T cell	CD3+	Macropage	CD68+
Helper T cell	CD3+ CD8-	M1-like macrophage	CD68+ CD206-
Cytoxic T cell	CD3+ CD8+	M2-like macrophage	CD68+ CD206+
Regulatory T cell	CD3+ CD8- FoxP3+	Neutrophil	CD15+
Memory helper T cell	CD3+ CD8- CD45RO+	Endothelium	CD31+
B cell	CD20+	MMP9+ cell	MMP9+ CD15-
classic DC type 2	CD1c + CD20-		

Antibodies used with clones between brackets (top). Markers used for defining specific cell populations are listed (bottom).

Tissue sections were deparaffinized, rehydrated and washed with demi water before heat induced antigen retrieval in EnVision™ FLEX target retrieval solution (pH 9, K8004, Agilent, Santa Clara, CA) for 10 min. Protein blocking was performed by covering the tissue in Akoya Antibody Diluent/Block (Akoya biosciences, MA). Primary antibodies were incubated for 1 h, followed by Polymer HRP Ms. + Rb (Akoya biosciences, MA) as a secondary antibody for 30 min. Visualization was done with tyramide signal amplification (TSA) using an Opal fluorophore (Akoya biosciences, MA) dissolved 1:50 in 1 X Plus Amplification Diluent (Akoya biosciences, MA). All incubations steps were performed at room temperature. Please see the Supplementary Table S1 in the Supplementary materials for a more detailed overview of the used reagents. To achieve a multiplex stain, featuring 6 markers on a single slide, this staining cycle was repeated five more times in series with a different Opal fluorophore for each marker. Finally, DAPI was used as a nuclear counterstain and slides were mounted with Fluoromount-G (0100–01, Southern Biotech, Birmingham, AL, United States).

### 2.4. Slide imaging and multispectral unmixing

Slides were scanned at 20× magnification using the PerkinElmer Vectra (Vectra 3.0.3; PerkinElmer, MA). Multispectral images were unmixed using spectral libraries and inForm Advanced Image Analysis software (inForm 2.4; Akoya biosciences, MA). Spectral libraries were built from images of single stained tissues for each reagent and a non-stained slide for the elastin autofluorescence.

### 2.5. Tissue and cell analysis

**Figure 1:**
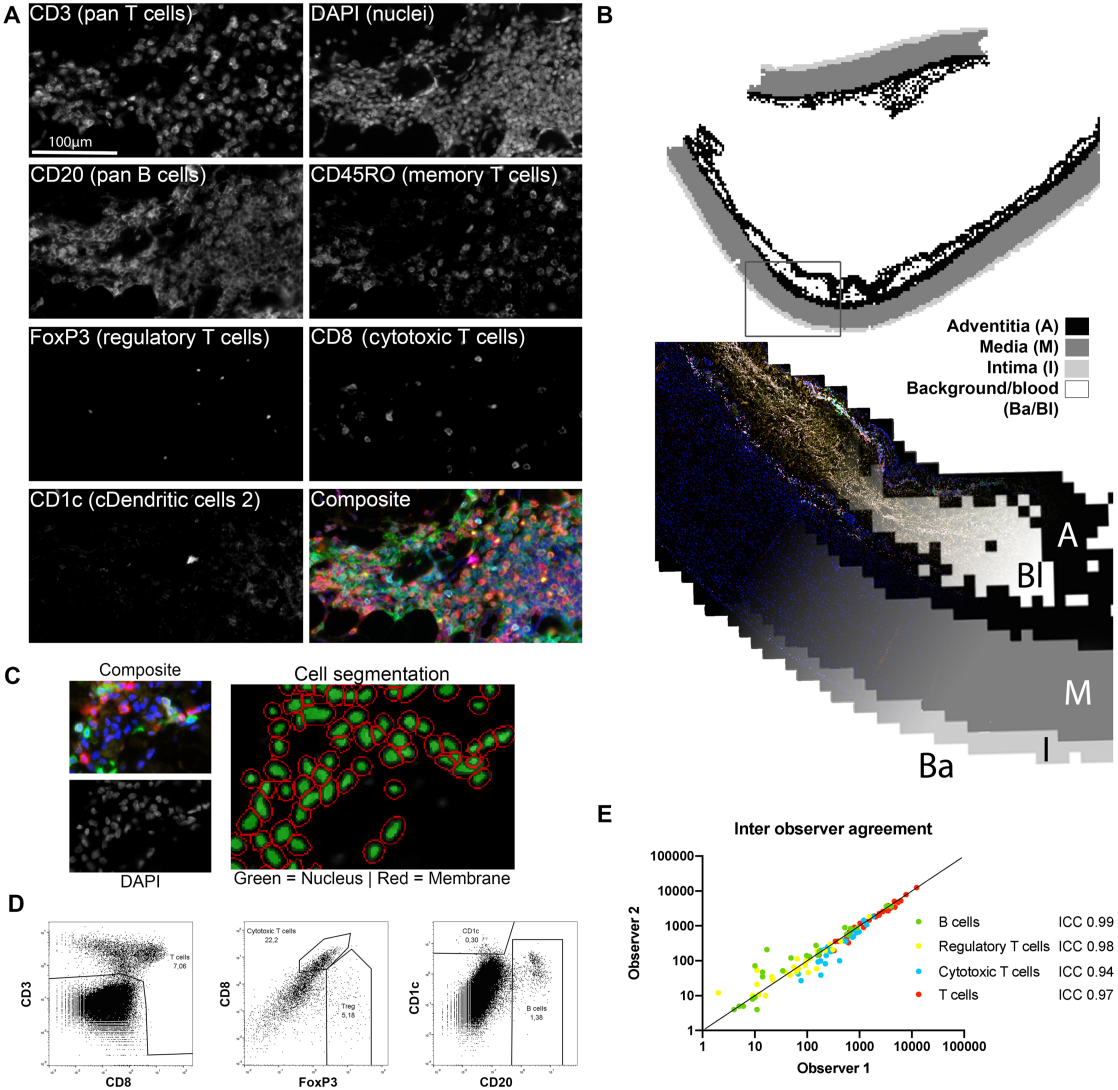
Image processing and data analysis methods. **(A)** All markers were stained consecutively and were simultaneously scanned with a fluorescence microscope and visualized with inForm. All separate channels of the adaptive immune cell panel (except autofluorescence) are shown to visualize the quality of the unmixing step. **(B)** inForm machine learning based tissue segmentation was used to distinguish the adventitia (A), media (M) and intima (I) and exclude the background (Ba) and blood (Bl) from the analysis. **(C)** Cell segmentation was based on identification of the nucleus on the DAPI signal and membrane identification on CD3, CD8 and CD20. **(D)** Single cell data visualized with FlowJo. Marker expression of the segmented single cells was assessed with a flowcytometry-like method. This resulted in cell populations that could be reproducibly gated as shown here for the adaptive immune cell panel. **(E)** Inter-observer agreement was excellent as measured with the intraclass correlation coefficient (ICC) thus cell population gating shows good reproducibility between observers (*n* = 27 for each cell type).

Whole slides consist of 2–4 tissue sections spanning multiple centimeters of aorta wall. The entire slide was scanned, resulting in up to 400 20×-views per subject. 20× magnification was chosen as it allowed accurate cell segmentation while simultaneously sampling a large aorta section which minimized sampling bias. Single cells were segmented using inForm Advance Image Analysis software which uses DAPI to identify cells and improves upon this segmentation using the membrane makers CD20 and CD3 when present. Artifact staining is very sparce and only present in necrotic areas void of cells. Excluding artifact staining in the analysis was possible by the DAPI based cell segmentation since only fluorescence associated with a cell is analyzed. Consequently, cell membranes without a nucleus -because it is outside of the section- will not be analyzed. This is most notable with large and irregularly shaped cells such as macrophages. Cell data (localization, tissue, phenotype, and marker expression data) and 20× view images were merged to form single flow cytometry standard files for each slide. These data were subsequently analyzed using FlowJo (Becton Dickinson, NJ). The tissue slides were divided into intima, media and adventitia based on the trained inForm tissue segmentation data (Figure 1B). Cells were phenotyped by gating as shown in Figure 1D. The expression of CD45RO and MMP9 is gradual as reported previously (37, 38), and does not result in a clearly separated positive population. Therefore, non-immune cells were used as the negative control population for CD45RO and MMP9 and cells with a higher expression of the marker compared to the negative control were marked as positive (see Supplementary Figure S1 for an example). FoxP3 was not analyzed in the non-dilated BAV ascending aorta group as these samples were obtained port-mortem, thus FoxP3 expression was not representative in this group. The inForm tissue segmenter was specifically trained on DAPI, elastin autofluorescence, CD20 and CD3 to recognize lymphoid infiltrates. Lymphoid infiltrates were defined as dense lymphocytic conglomerates where both T- and B-lymphocytes are physically touching. Subsequently, these regions were scored as tertiary lymphoid structures (TLS) by a pathologist in training based on the presence of separate B and T cell zones, venules or germinal centers.

Samples were graded on presence and magnitude of atherosclerosis and media degeneration using the AHA classification and the most recent consensus statement by the AECVP/SCVP (39, 40).

### 2.6. Nearest neighbor analysis

Nearest neighbor analysis was performed on M1-like macrophages (CD68+ CD206-) and endothelial cells (CD31+ CD68-) located in the media and adventitia of all aortas as phenotyped by inForm cell phenotyper. All data belonging to one slide were merged in order to find nearest neighbors on adjacent tiles. Slides that contained less than 150 cells of one phenotype were excluded because of a high chance for a sample error. An adapted version of the Akoya Biosciences ‘phenoptr’ (41) nearest neighbor analysis was performed, finding the median minimal distance between M1-like macrophages and endothelial cells.

### 2.7. Statistical analysis

Statistical analysis was performed with SPSS for Windows (IBM Corp, 2017. IBM SPSS Statistics for Windows, Version 25.0. Armonk, NY: IBM Corp). Visualization of results was done with PRISM 8 (Graphpad, GSL Biotech LLC, CA). Continuous data were expressed as mean ± standard deviation (SD), or in case of non-Gaussian distribution median (interquartile range) (IQR). The non-Gaussian distributed variables were compared between two groups with the Kolmogorov–Smirnov test. Testing between three groups was performed with the independent Kruskal-Wallis test adjusted with Bonferroni correction for multiple testing. Binary variables were tested for differences using the Fisher exact test. Interobserver variability was calculated with the intraclass correlation coefficient (ICC). Correlations between continuous non-Gaussian distributed variables were studied with Kendall’s tau because of low numbers per group. *p* < 0.05 was considered statistically significant.

## 3. Results

### 3.1. Patient baseline characteristics

Bicuspid aortic valve ascending aorta samples were collected and divided into 3 groups: non-dilated, dilated and dissection. The diameter of dilated and dissected aortas did not differ significantly (Table 2). The median time between acute aortic dissection, defined as onset of symptoms, and surgery was 4.4 (2.5–15.5) hours (Table 2).

**Table 2:** Baseline characteristics of the study cohort (*n* = 27).

	Non-dilated (%) *n* = 8	Dilated (%) *n* = 15	Dissection (%) *n* = 4	*p* value
Male sex	7 (88)	14 (93)	2 (50)	0.172
Age (year)	50.5 (47–54)	57 (52–64)	67 (43–70)	0.098
Time from symptoms to surgery (hours)	n/a	n/a	4.4 (2.5–15.5)	
Diameter (mm)	33 (23–38)	48 (43–55)	54 (47–64)	0.002
	33 (23–38)	48 (43–55)		0.007
	33 (23–38)		54 (47–64)	0.003
		48 (43–55)	54 (47–64)	1.0
Valvulopathy				
Aortic stenosis	1 (13)	7 (47)	1 (25)	0.54
Aortic regurgitation	1 (13)	2 (13)	0 (0)	0.74
Combined	1 (13)	5 (33)	1 (25)	0.55
Medical history				
Hypertension	3 (36)	5 (33)	1 (25)	0.64
Previous surgery	0 (0)	0 (0)	0 (0)	n/a
Medication				
RAS inhibition*	2 (25)	2 (13)	0 (0)	1
Other anti-hypertensive drugs	1 (8)	3 (20)	1 (25)	0.807

Data are shown as *n* (%) or median ± interquartile range, statistical significance calculated with Kruskal Wallis test or Fisher’s exact test. *Renin-angiotensin system inhibition.

### 3.2. Quality of *in situ* single cell phenotyping in quantitative multiplex immunohistochemistry

Two antibody panels to identify cells of either the innate or the adaptive immune system were developed (Table 1). These panels resulted in eight colors from 6 antibodies markers, DAPI and elastin autofluorescence on a single slide. Since all antibodies were stained on the same slide, colocalization was excellent as shown in Supplementary Figure S2 of the Data Supplement. After unmixing of the eight overlapping fluorescence spectra bleed-through was absent (Figure 1A; Supplementary Figure S3A in the Data Supplement).

After unmixing, images were refined in a three-step process to extract whole slide *in situ* single cell phenotype data. First, the inForm tissue segmenter, based on supervised machine learning, was employed to distinguish the intima, media and adventitia layers as shown in Figure 1B. This allowed for separate analysis of the different layers and exclusion of hematomas caused by the surgery or dissection where circulating peripheral blood cells are present (Figure 1B). Tissue segmentation information was used for a layer specific analysis of vascular tissue.

In the second step, cells were virtually dissociated with the inForm cell segmentation tool (Figure 1C). Finally, cells were divided in populations based on marker expression as assessed by a flowcytometry-like method (Figure 1D; Supplementary Figure S4 in the Data Supplement). The interclass correlation coefficient (ICC) of two blinded observers was excellent (Figure 1E), demonstrating robustness of this analysis approach. This semi-automated approach allows analysis of entire slides decreasing sampling error. In this study, the mean number of 20×-magnification views analyzed per subject was 228 ± 107. Together, these data show that the method employed is suitable for *in situ*, localized, single cell analysis of entire tissue slides with little inter-observer variability and IHC artefacts.

### 3.3. Large numbers of lymphocytes, organized in tertiary lymphoid structures, are observed in the adventitia of dilated compared to non-dilated bicuspid aortic valve ascending aortas

The adaptive immune system is studied by markers discriminating B cells, T cells and various subsets thereof (Table 1). Distinguishing these subsets provides insight in T cell function and activation state. Furthermore, type 2 classic dendritic cells (cDC2), which interact with and present antigen to helper T cells, can be identified (29).

**Figure 2:**
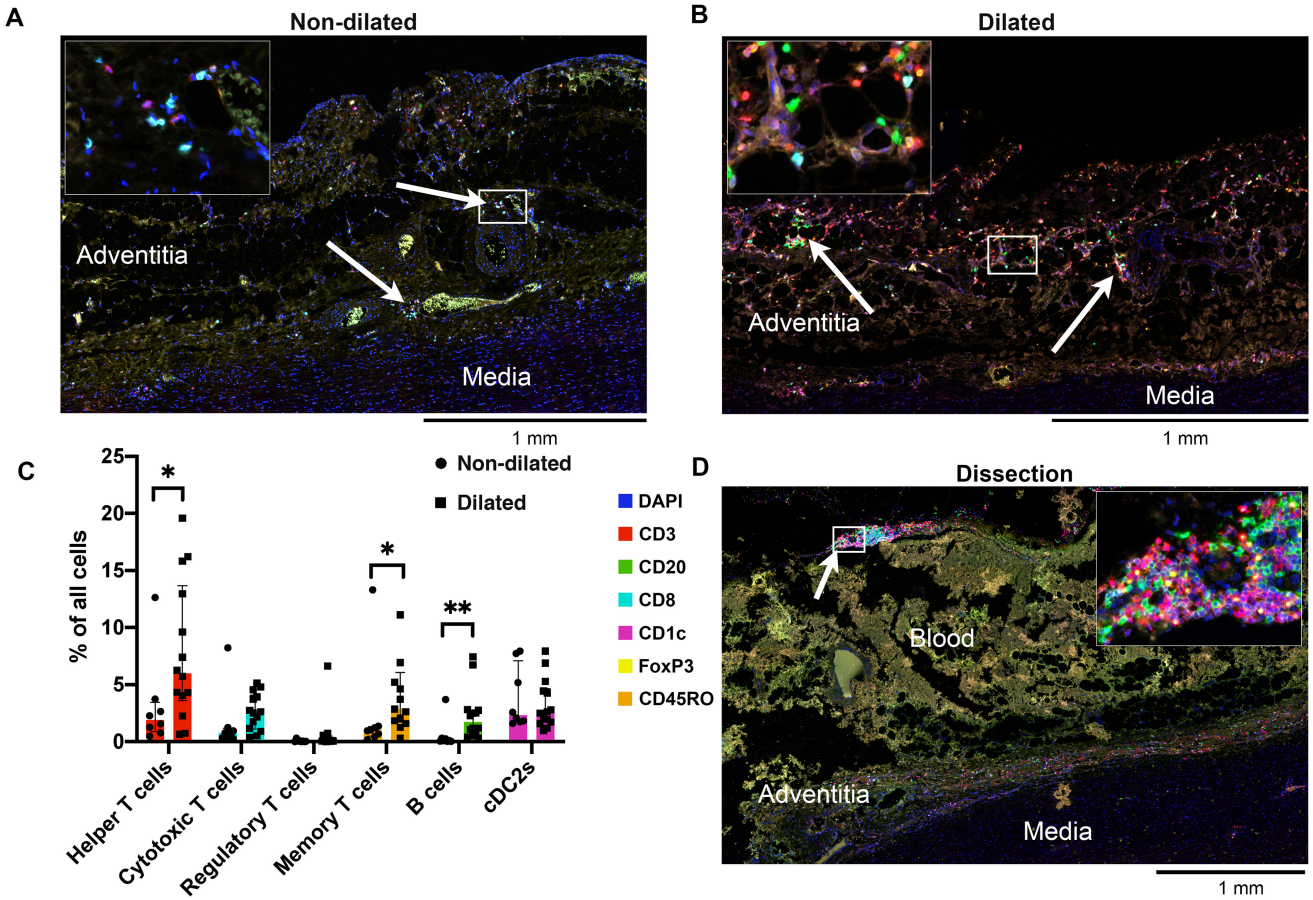
Adventitia of dilated aortas showed increased numbers of lymphocytes. **(A)** Representative cross-section of the adventitia of a non-dilated BAV ascending aorta (*n* = 13). Note the helper T cells, cytotoxic T cells and cDC2s which are mainly situated around the vasa vasorum (arrows). **(B)** Representative cross-section of the adventitia of a dilated aorta (*n* = 15). Note the strong increase of T cells and the appearance of a significant number of B cells (arrows). **(C)** Quantification of adaptive immune system cells in the adventitia of non-dilated (*n* = 8) and aneurysmatic (*n* = 15) BAV aortas, total analyzed 20×-views = 6,318. Data shown as median ± inter-quartile range, *p < 0.05, **p < 0.01 as calculated with the independent Kolmogrov-Smirnov Test. **(D)** Representative cross-section of the adventitia of a dissected BAV ascending aorta (*n* = 4). The adventitia has largely been destroyed by the force of the entering blood. However, a large number of lymphocytes can be seen scattered close to the media and at the edge of the adventitia (arrow).

The adventitia of dilated BAV ascending aortas was compared to non-dilated BAV samples (Figures 2A,B). The adventitia of dilated samples contained 4-times more lymphocytes and 25-times more B cells when compared to non-dilated samples (Figure 2C). Helper T cells were the most abundant lymphocyte subset making up 12.42 (5.89–19.58)% of all cells in the adventitia. The CD4/CD8 ratio in dilated aortas was 2.52 (1.76–4.44) compared to 1.82 (1.52–4.67) non-dilated samples.

Helper T cell/B cell interaction is a central event in the adaptive immune response, a positive correlation between these cell types is observed in dilated BAV ascending aortas (*r* = 0.411 (CI 0.087–0.671), *p* = 0.007). The number of CD45RO+ memory T cells was significantly higher in the dilated compared to the non-dilated group (*p* < 0.01). Finally, regulatory T cells were rarely observed in the adventitia of dilated BAV ascending aortas (Figure 2C).

Visually, dissected BAV ascending aortas also show the presence of T and B lymphocytes as shown by a representative sample in Figure 2D. The adventitia in dissected aortas was too disrupted to perform a quantitative analysis.

**Figure 3:**
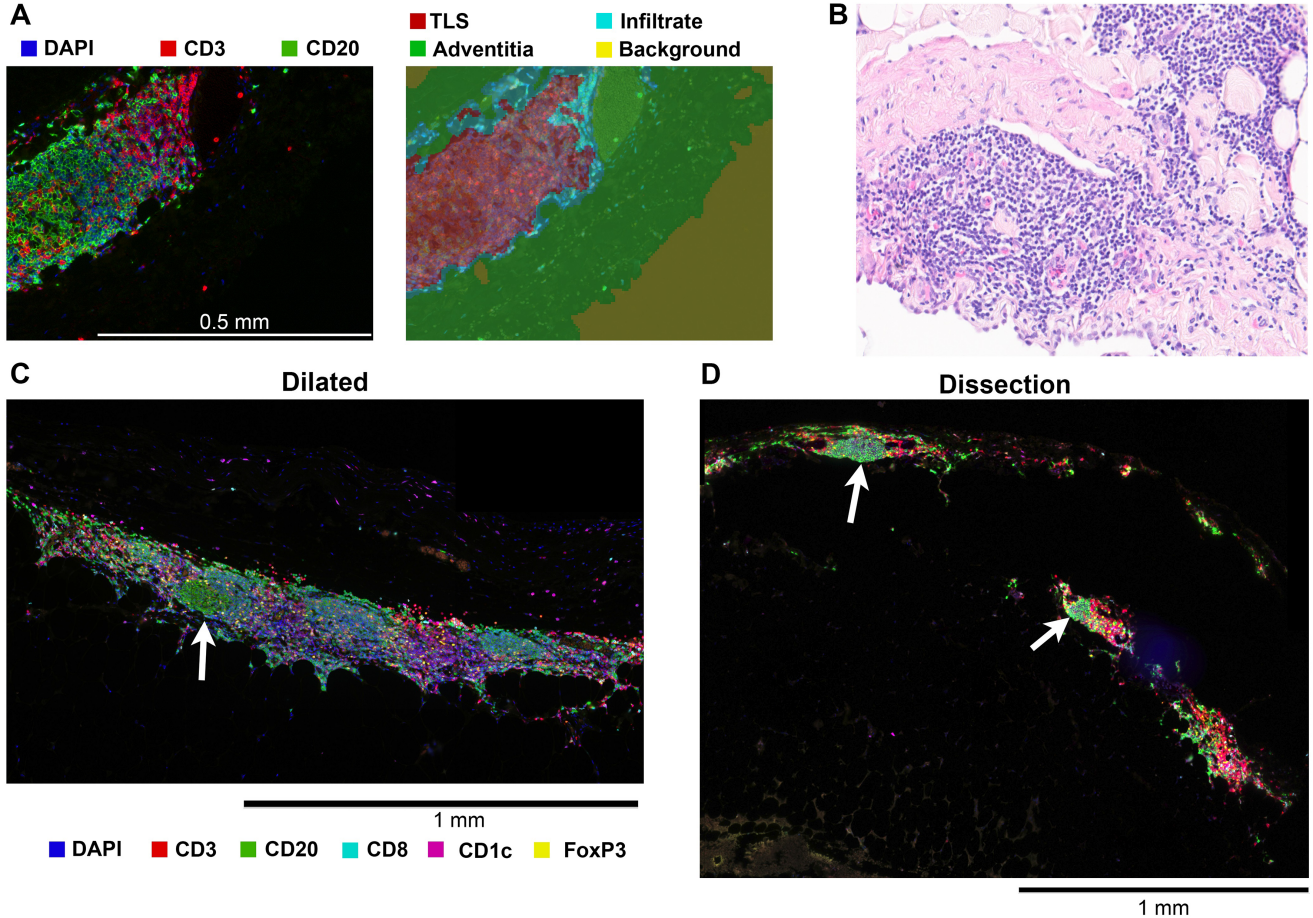
Tertiary lymphoid structures (TLS) were only identified in the adventitia of dilated aortas and dissections. **(A)** Left, a 20x magnified image of a tertiary lymphoid structure showing the separate B and T cell zones and a germinal center. Right, result of tissue segmentation of this 20x magnified image with a machine learning algorithm. **(B)** 20x magnification brightfield hematoxylin and eosin stain of a TLS in a dilated aorta. Plasma cells, not stained by markers in our two antibody panels, are visible. **(C)** Representative image of a TLS found in the adventitia of an aneurysmatic aorta, germinal center indicated with an arrow. **(D)** Although most dissected BAV ascending aortas had a non-representative adventitia due to the disruption of the entering blood, TLS with germinal centers (white arrow) were observed.

Multiplex IHC allowed for whole slide quantification of immune cell subsets while also maintaining morphological information. Through this *in situ* approach, organized structures of T- and B-cells and cDC2s in the adventitia were observed. These structures contain T and B cell zones, germinal centers and venules (Figure 3). These formations were classified as tertiary lymphoid structures (TLS) based on these observations. To quantify TLS in an unbiased way, a machine learning algorithm was used (Figure 3A). TLS were present in the adventitia of 50% of dilated BAV samples whereas in non-dilated BAV ascending aortas no TLS were present. The average number of TLS per slide was 2.6 and the average distance between a TLS and the aorta media was 242 μm. TLS were also observed in dissected samples however these were not quantified as the destroyed adventitia morphology did not allow robust analysis (Figures 2D, 3D).

Together these data indicate a chronic activation of the adaptive immune system.

### 3.4. Adaptive immune cells are sparce in the aorta media

**Figure 4:**
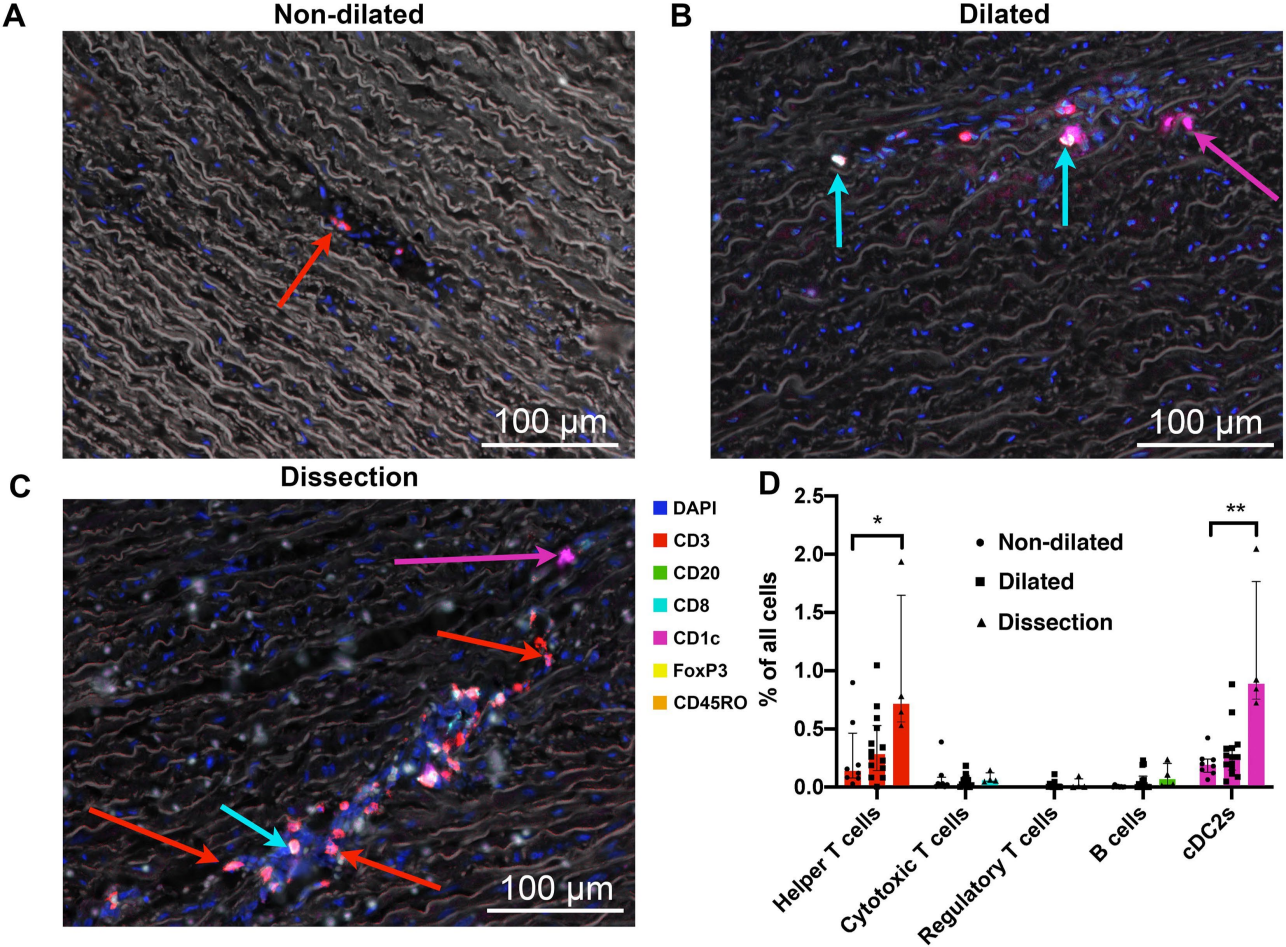
The media of dissected aortas showed an increased number of helper T cells and cDC2s. **(A)** Representative cross-section of the media of a non-dilated BAV ascending aorta (*n* = 8). Sparse helper T cells (red arrow) were found in outer 2/3th of the media often in blood vessels. **(B)** Representative cross-section of the media of a dilated BAV ascending aorta (*n* = 15). A slight increase in number of T cells, now extravasated into the tissue, was observed in the media of dilated aorta samples. Cytotoxic T cells are indicated with a cyan arrow, cDC2 cells are indicated with a magenta arrow. Note the decrease of elastin density and elastin fiber breaks. **(C)** Infiltration of helper T cells (red arrow), and the occasional cytotoxic T cell (cyan arrow) in the aortic media of dissected aortas (*n* = 4). We also observed an increased number of cDC2s (magenta arrow) in these samples. Note the decrease of elastin density and elastin fiber breaks. **(D)** Quantification of adaptive immune system cells in the media of non-dilated (*n* = 8, dots), dilated (*n* =15, squares) and dissected (*n* = 4, triangles) BAV aortas. Data shown as median (interquartile range), *p < 0.05, **p < 0.01 as calculated with the independent Kruskal-Wallis Test adjusted with Bonferroni correction for multiple tests. Total analyzed 20x-views = 6318.

Deterioration of the integrity of the aortic media is generally seen as the key process leading to a vulnerable aorta. Adaptive immune cells in the aorta media were sparce across all groups (Figure 4). Significant differences in the number cDC2s and helper T cells were seen between non-dilated and dissected aortas. We found these helper T cells and cDC2s only in areas surrounding the vasa vasorum (Figure 4). Media degeneration was observed in 27% of non-dilated samples and 20% of dilated samples and was mild in all but one case. This is in line with previous studies.

### 3.5. Dilated and dissected aortas show increased numbers of M1-like macrophages

With a second multiplex IHC panel the number of CD15 neutrophils and CD68 macrophages, as well as their polarization based on presence or absence of CD206, were assessed (Table 1). Furthermore, the expression of one of the important metalloproteinases (MMP) in aortic disease, MMP9, was studied to investigate whether this specific MMP contributed to matrix vulnerability in BAV aortopathy.

**Figure 5:**
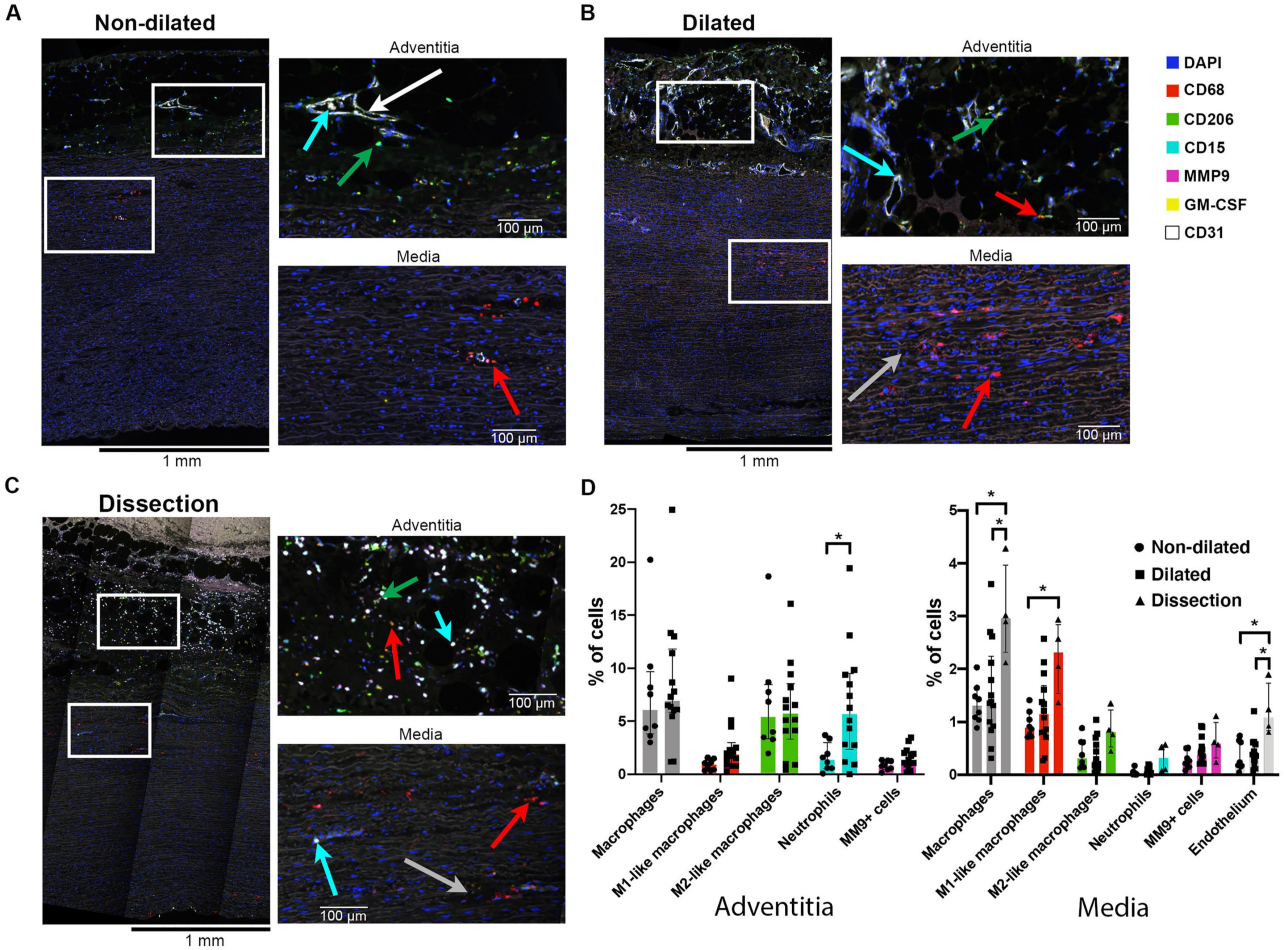
Dilated aortas and dissections showed increased numbers of macrophages in the aorta media. **(A)** Full thickness sample of a non-dilated BAV ascending aorta (*n* = 8). Note sparse M1-like macrophages (red arrow) in the media, closely situated to the sparse *vasa vasorum* (endothelium in white). The adventitia showed mainly M2-like macrophages (green arrow). Neutrophils were found intra-vascular (cyan arrow). **(B)** Full thickness sample of a dilated BAV ascending aorta (*n* = 15). Note the increase in M1-like macrophages and some disruption of the media elastin fibers (grey arrow). **(C)** Full thickness sample of a dissected BAV ascending aorta (*n* = 4). Note the increase in M1-like macrophages (red arrow) in the media and strong infiltration of neutrophils (cyan arrow) in the adventitia alone. **(D)** Quantification of innate immune system cells in the media and adventitia of non-dilated (*n* = 8), dilated (*n* = 15) and dissections (*n* = 4), total analyzed 20×-views = 5,931. Data shown as median ± inter-quartile range, *p < 0.05, **p < 0.01 as calculated with the independent Kruskal-Wallis Test adjusted with Bonferroni correction for multiple tests.

The adventitia of dilated BAV ascending aortas contained 1.6 times more CD68 macrophages compared to non-dilated samples (Figure 5D). The tunica media showed a gradual increase in numbers of macrophages from non-dilated to dilated and dissected aorta samples (Figure 5). The majority of media macrophages was M1-like in contrast to the adventitia macrophages that were mostly M2-like. However, the M1/M2 ratio of both the media and adventitia macrophages did not show significant change between non-dilated and dilated BAV aortas.

GM-CSF, a pro-inflammatory cytokine implicated in the onset of aortic dissections (42), was observed in vascular smooth muscle cells (VSMC) and media infiltrating immune cells of dissections and some dilated aortas (Supplementary Figures S7B–D in the Data Supplement). Non-dilated aortas rarely showed GM-CSF positive cells (Supplementary Figure S7A in the Data Supplement).

We did not observe differences in expression of (pro-) MMP9 between the three groups, this could indicate that this specific metalloproteinase did not contribute to BAV aortopathy (Figure 5D).

Although the adventitia of dissections was disrupted, visually a strong increase in neutrophils was observed compared to non-dilated samples (Supplementary Figure S6 in the Data Supplement).

Together this data suggests an association between number of macrophages in the tunica adventitia and media and aortopathy. GM-CSF might play a role in the increased numbers observed. MMP9 could not be identified as an important effector molecule.

### 3.6. Dilated bicuspid aortic valve ascending aortas do not show increased atherosclerotic features compared to non-dilated samples

The tunica intima was investigated for signs of atherosclerosis to rule-out atherosclerosis as the origin of the observed immune cells. In this cohort, no differences are observed in immune cell numbers in the intima between non-dilated, dilated and dissected aortas (Supplementary Figure S5 in the Data Supplement). The combined number of immune cells was as low as 8.1% of all cells in the intima. M1-like macrophages were encountered most often at 4.6% of all cells. These findings suggest that atherosclerosis does not play a role in the development of ascending aorta aneurysms and dissections in patients with BAV.

### 3.7. *In situ* phenotyping of cells allows for nearest neighbor analysis to asses cell migration

Multiplex IHC allows identification of cell types while maintaining information about their specific location within the sample. This allowed an explorative analysis.

**Figure 6:**
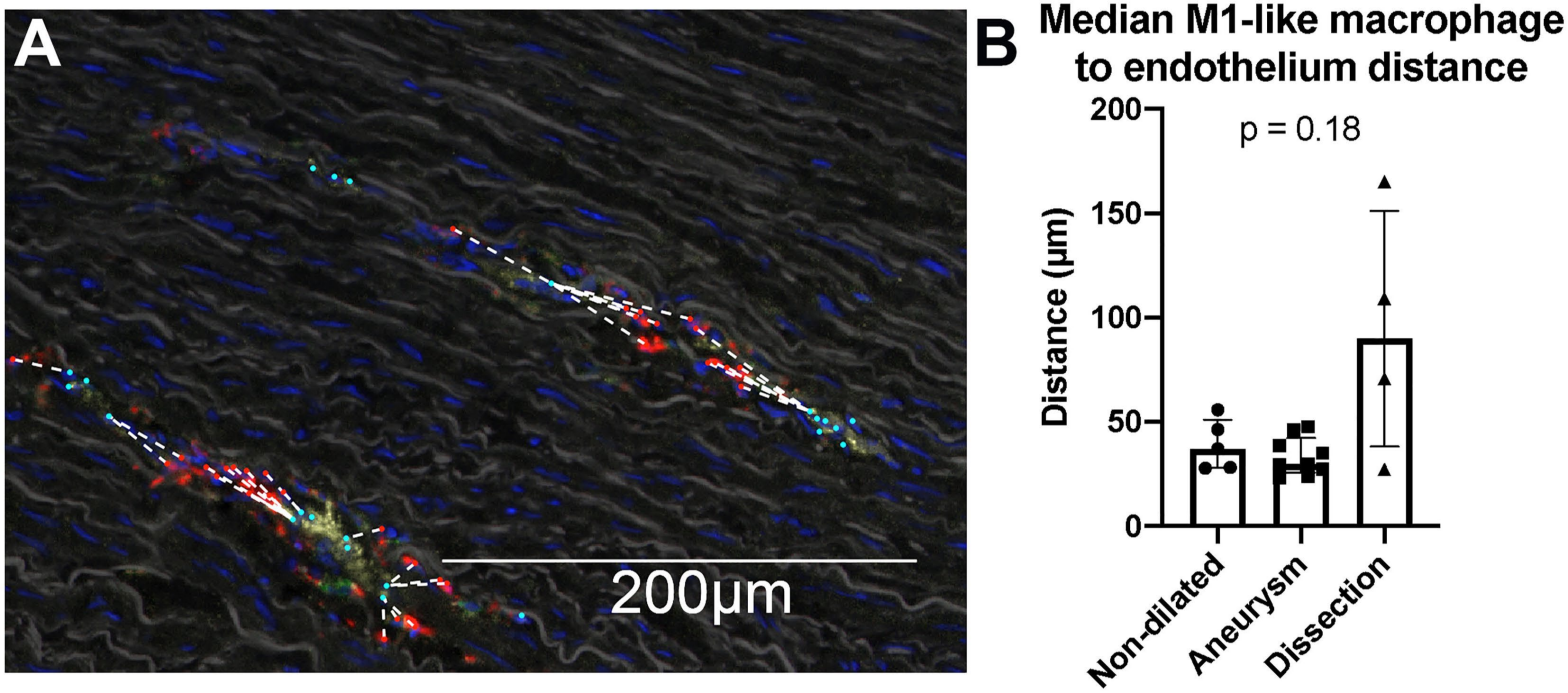
Nearest neighbor analysis of the distance between M1-like macrophages and endothelium did not show macrophages in dissections closer to the *vasa vasorum*. **(A)** Visualization of the nearest neighbor analysis on a single 20× view. M1-like macrophages are marked in red, endothelium in cyan, the distance between a M1-like macrophage and the closest endothelial cell was marked with a dashed white line. Cells are only phenotyped in the presence of a DAPI stained nucleus, which is an important quality control mechanism to prevent analysis of artefact staining. CD68 staining (in red) of the irregular membrane of macrophages can be seen without the cells’ nucleus in the section, consequently these signals are not analyzed as a cell. Note that the full analysis was done on a whole slide level, not on separate 20× views. **(B)** Quantification of the median distance between M1-like macrophages and endothelium. We do not observe a significant difference in this distance between non-aneurysmatic (*n* = 5), aneurysmatic (*n* = 9) and dissected (*n* = 4) samples as calculated with the Kruskal Wallis test. Data shown as median ± inter-quartile range.

To study whether the increase in macrophages in dissected compared to non-dilated samples can be attributed to monocyte recruitment from the circulation, a nearest neighbor analysis was performed to study the distance between macrophages and the nearest vasa vasorum (Figure 6A). If cells would have been recruited based on an acute event (i.e., dissection), it would be expected that these cells were found much closer to the nearest vasa vasorum compared to non-dilated or dilated samples (43). We did not observe differences in the median distance between macrophages and endothelial cells for all groups (Figure 6B). This observation might indicate that the acute dissection did not yet result in extravasation of additional monocyte/macrophages and that the number of macrophages observed were likely present before the dissection.

## 4. Discussion

Bicuspid aortic valve is a highly prevalent congenital heart defect with a significant risk of developing aortopathy and AAS (10). The clinical task of preventing high-mortality complications such as an aortic dissection is difficult and likely leads to both over and under treatment. An incomplete understanding of the underlying mechanisms leading to aorta dilatation and acute aortic dissection challenges recognition of the vulnerable aorta. This study shows abundant immune cells in the adventitia of dilated ascending aortas in patients with BAV. These immune cells are organized in tertiary lymphoid structures, suggesting chronic and non-resolving inflammation.

Previously, inflammation in BAV aortopathy was studied using conventional hematoxylin–eosin staining or IHC with macrophage markers such as CD68 (21–23). The majority of these studies did not show significant differences in immune cell numbers between non- and dilated aortas of patients with a BAV. Moreover, many BAV aorta aneurysms appear normal in terms of media matrix composition including lack of cystic medial degeneration (44, 45). Yet, other studies described neovascularization and low grade inflammation in aneurysmatic and dissected BAV aortas (23, 25). In contrast to BAV, increased immune cells are well established in tricuspid valve ascending aortic aneurysms (46, 47).

We implemented a novel whole slide quantitative multiplex IHC method that allowed investigation of diverse immune cells in aortic tissue in a highly sophisticated manner. We employed a quantification method with remarkably good interobserver agreement and thus minimized observer bias. Furthermore, we minimized sampling bias by quantifying immune cells on an average of over 200 20×-magnification views per subject. Therefore, this novel method might be more appropriate to discover biologically important differences in immune cell composition (33, 48).

Bicuspid aortic valve aortopathy develops through unique genetic factors and flow pattern mechanisms (6, 10–13, 49). BAV aorta aneurysms are characterized by disruption of elastin fibers, VSMC death, and loss of matrix integrity, all forms of tissue damage (5, 10, 50, 51). This tissue damage is more specifically the result from abnormal flow patterns (13, 52), MMP2 upregulation (6, 22), VSMC oxidative stress and erroneous collagen and elastin biosynthesis, cross-linking and microarchitecture (50, 53). These combined processes lead to the formation of Damage-associated molecular patterns (DAMPs). DAMPs are mostly TLR-ligands and activate resident immune cells. As a result, circulating leukocytes are recruited to these damaged regions (54). For example, TLR-4 ligands such as heat-shock protein (hsp) 70 are induced during vessel wall damage from acute hypertension (51). Immune cell activation through pattern recognition receptors might explain the increased number of immune cells observed in this study.

Recruited lymphocytes organize into clusters where antigens are continuously presented by professional APCs. In general, only when a specific antigen is recognized by a lymphocyte the lymphocyte clusters will develop into a TLS. Previous research has shown that a nonspecific stimulus of the TLR-4 receptor is insufficient to form germinal centers within these TLS (55, 56). Therefore, tissue damage may explain the presence of lymphocytic cell clusters, yet an alternative explanation for the formation of germinal centers is needed.

Tertiary lymphoid structures were observed surprisingly often in the adventitia of dilated aortas. In contrast, no TLS formation was observed in non-dilated aortas. TLS can be formed at sites of infection or chronic immune stimulation and are associated with auto-inflammatory disorders. The presence of TLS and their association with tissue damage in chronic diseases has led to the suggestion that TLS are important inductive sites for T cells and antibodies that contribute to pathology (57, 58). In atherosclerosis, TLS in the adventitia are associated with infiltration of macrophages and T cells in the media and media erosion (59). This finding has clear similarities with what we observe. However, we do not find any differences in immune cell numbers in the intima, which indicates that atherosclerosis is not the underlying mechanism in BAV aortopathy and dissections, which has been shown earlier as well (60). Recent work by Gu et al. identified auto-reactive T cells to elastin fragments in the peripheral blood of patients with thoracic aneurysm disease (61). A similar auto-antigen might be responsible for the immune cell infiltrate and TLS formation observed in this study.

However, it should be noted that the activation of the adaptive immune system is highly context dependent and influenced by environmental, microbiome and life-style factors (62). Therefore, it is probable that the presence of such auto-antigen will only lead to immune system engagement in a specific tissue with specific factors present, which could explain why other elastin containing tissues are not affected in these patients. It should be noted that our study is not designed to provide evidence for specific mechanisms underlying adaptive immune cell activation.

The main finding of the innate immune system panel is the increase in macrophages in the tunica adventitia of dilated BAV ascending aorta compared to non-dilated samples. Macrophages in the tunica media are mainly M1-like macrophages which are associated with tissue damage. Furthermore, there is a correlation between M1 macrophages and helper T cells in de tunica media. These helper T cells might further activate macrophages to produce pro-inflammatory cytokines and chemokines such as GM-CSF, as indeed was observed in this study and in previous pre-clinical and clinical work (42). This complex interaction between innate and adaptive parts of the immune system could play a role in aorta vulnerability, especially when this interaction is deregulated.

Some limitations need to be acknowledged. First, the sample size of our groups is limited. This is in part mitigated by analyzing and quantifying entire slides. This totaled to an average of over 200 20×-views per sample. This minimizes the sampling bias and is therefore a better representation of the ongoing processes in BAV aortopathy. Our findings are only representative of the BAV subpopulation of patients with aortopathy. These results cannot be extrapolated to tricuspid aortic valve patients and patients with Marfan syndrome given the important distinctions between these aortopathy subpopulations. However, comparable results could be expected based on earlier work (46, 47) and investigation of these subpopulations would therefore be an interesting area of future research. Lastly, our findings are only descriptive of ongoing processes in human aorta dilatation and dissections. To prove a causal relationship animal testing is required. However, the currently used mouse models might struggle to represent human aneurysms and dissections, since their disease development is so rapidly that the low-grade and chronic inflammation has no time to develop (63, 64); in atherosclerotic mice it takes 32 week for TLS to develop (59).

Dissection samples were challenging to analyze due to the tissue damage from the acute event. This tissue damage might have led to immune cell recruitment and therefore inflated cell numbers. However, in various models of acute injury, numbers of T cells, macrophages and myeloid DCs are not increasing within the first 12 h after injury (65–68). Since the median time from first symptoms of dissection to surgery was 4.4 h, it is likely that these cells were present before the acute event. Our nearest neighbor analysis supports this proposition. Taking these limitations into account, the results from dissection samples should be interpreted with caution.

In summary, degenerative processes attributing to dissection in BAV aortopathy are numerous. Genetic factors, embryonic origin of VSMC and pathogenic flow patterns which subsequently alter VSMC differentiation and function take a central place in our current understanding of ascending aorta aneurysms in BAV. Here we introduce a novel analyses method to investigate immunological processes in vascular disease. With this method, chronic immune activation in BAV aortopathy was visualized, expanding on the limited knowledge that is available on this subject. The number of immune cells present in the adventitia of dilated aortas of patients with BAV is much larger than expected.

We cannot distinguish between a bystander effect or a destructive function of the immune system and the cause-effect. The unexpected presence of TLS does suggest an active role of the immune system. It could be hypothesized that aberrant activation of the adaptive immune system contributes to the process of aortic vulnerability, alongside genetic factors and flow-mediated mechanisms. Identification of the key inflammatory regulators as the final step toward aortic dissection opens up new approaches to disease management. Inflammatory regulators could lead to new biomarkers identifying patients at high risk of aortic dissection as well as new medical options to prevent aorta vulnerability by suppressing this tissue damaging inflammation. Further investigation into the role of the immune system in the pathogenesis of AAS in BAV is warranted and could lead to new diagnostic or therapeutic avenues.

## Data availability statement

The raw data supporting the conclusions of this article will be made available by the authors, without undue reservation.

## Ethics statement

The studies involving human participants were reviewed and approved by CMO Radboudumc. Written informed consent for participation was not required for this study in accordance with the national legislation and the institutional requirements.

## Author contributions

AS conception and design, acquisition, analysis, interpretation of data, and drafting for the work. KC statistics and assistance in writing. MG and LW assistance in analysis and interpretation of data. RH and GG providing aorta tissues. NG acquisition of data. KH assistance in interpretation of data. MS critical appraisal of concept. MD providing aorta tissues and critical appraisal of concept. IV and RK final supervision in concept, design, and writing. All authors contributed to the article and approved the submitted version.

## Funding

This work was supported by a European Research Council starting grant [ERC-2014-StG-336454-CoNQUeST], a Toegepaste Technische Wetenschappen - Nederlandse Organisatie voor Wetenschappelijk Onderzoek (TTW)-(NWO) open technology grant [STW-14716] and a SCAN consortium: European Research Area - CardioVascualar Diseases (ERA-CVD) grant [JTC2017-044].

## Conflict of interest

The authors declare that the research was conducted in the absence of any commercial or financial relationships that could be construed as a potential conflict of interest.

## Publisher’s note

All claims expressed in this article are solely those of the authors and do not necessarily represent those of their affiliated organizations, or those of the publisher, the editors and the reviewers. Any product that may be evaluated in this article, or claim that may be made by its manufacturer, is not guaranteed or endorsed by the publisher.

## Abbreviations

AAS: acute aortic syndrome
APC: antigen presenting cell
BAV: bicuspid aortic valve
cDC2: classic dendritic cell type 2
DAMP: damage associated molecular pattern
DC: dendritic cell
FFPE: formalin fixed and paraffin embedded
GM-CSF: granulocyte-macrophage colony stimulating factor
Hsp: heat shock protein
ICC: intraclass correlation coefficient
IHC: immunohistochemistry
IQR: interquartile range
MMP: matrix metalloproteinase
TLS: tertiary lymphoid structure
TLR: toll-like receptor
VSMC: vascular smooth muscle cell
