# Supplementary material for: Adventitial adaptive immune cells are associated with ascending aortic dilatation in patients with a bicuspid aortic valve
Source: Front Cardiovasc Med. 2023 Mar 28;10:1127685. doi: 10.3389/fcvm.2023.1127685 (PMC10086356; doi:10.3389/fcvm.2023.1127685)
Supplement: Supplementary file 1 [file Data_Sheet_1.PDF]

Supplementary information

Adventitial adaptive immune cells are associated with ascending aortic dilatation in patients with a bicuspid aortic valve

Alexander H.J. Staal MD PhD1#, Kimberley R.G. Cortenbach MD PhD1#, Mark A.J. Gorris MSc PhD1, Lieke L. van der Woude MD1,4, Mangala Srinivas PhD1, Robin H. Heijmen MD PhD2, Guillaume S.C. Geuzebroek MD PhD2, Nimrat Grewal MD PhD3, Konnie M. Hebeda MD PhD4, I. Jolanda M. de Vries PhD1 Marco C. DeRuiter PhD6, Roland R.J. van Kimmenade MD PhD*7

1 Department of Tumor Immunology, Radboud Institute for Molecular Life Sciences, Radboud University Medical Center, Nijmegen, the Netherlands
2 Department of Cardiothoracic Surgery, Radboud University Medical Center, Nijmegen, the Netherlands
3 Department of Cardiothoracic Surgery, Leiden University Medical Center, Leiden, the Netherlands
4 Department of Pathology, Radboud University Medical Center, Nijmegen, the Netherlands
6 Department of Anatomy and Embryology, Leiden University Medical Center, Leiden, the Netherlands
7 Department of Cardiology, Radboud University Medical Center, Nijmegen, the Netherlands

# authors contributed equally

Antibodies

Target antigen	Vendor	Catalog #	Working concentration	Lot #	Persistent ID / URL
CD3 (SP7)	Thermo-Fisher	RM-9107	1:400	9107S1903A	https://www.thermofisher.com/order/catalog/product/RM-9107-R7#/RM-9107-R7
CD8 (CD8/144B)	Dako	M7103	1:1600		
CD20 (L26)	Thermo-Fisher	MS-340-S	1:300	340S1705A	https://www.thermofisher.com/order/catalog/product/MS-340-R7#/MS-340-R7
CD1c (2F4)	Abcam	Ab156708	1:150		https://www.abcam.com/cd1c-antibody-oti2f4-ab156708.html
FoxP3 (236A/E7)	eBioscience	14-4777-82	1:300		https://www.thermofisher.com/antibody/product/FOXP3-Antibody-clone-236A-E7-Monoclonal/14-4777-82
CD45RO (UCHL-1)	Thermo-Fisher	MS-112-p	1:3000	112p1712B	https://www.thermofisher.com/order/catalog/product/MS-112-P#/MS-112-P
CD68 (PG-M1)	Dako	M087601	1:200	20029531	
CD206 (CL038+)	Sigma	AMAB90746	1:2500	02683	https://www.sigmaaldrich.com/catalog/product/sigma/amab90746?lang=en&region=US
CD15 (MMA)	BD Biosciences	559045	1:600	8004667	https://www.bdbiosciences.com/us/applications/research/stem-cell-research/cancer-research/human/purified-mouse-anti-human-cd15-mma/p/559045
CD31 (JC70A)	Dako	M0823	1:800	0054859	
MMP9 (polyclonal)	Atlas antibodies	HPA001238	1:600	D106525	https://www.atlasantibodies.com/products/antibodies/primary-antibodies/triple-a-polyclonals/mmp9-antibody-hpa001238/
GM-CSF (polyclonal)	Sanbio	PP1101P1	1:200	0905M030RB G2414 cv	https://www.origene.com/catalog/antibodies/primary-antibodies/pp1101p1/gm-csf-csf2-rabbit-polyclonal-antibody

Other

Description	Source / Repository	Persistent ID / URL
Silane coated glass slides	New Silane III, MUTO PURE CHEMICALS, Japan	
EnVision™ FLEX target retrieval solution	K8004, Agilent, Santa Clara, CA	
Akoya Antibody Diluent/Block	Akoya biosciences, MA	
Polymer HRP Ms + Rb	Akoya biosciences, MA	
Plus Amplification Diluent	Akoya biosciences, MA	

Fluoromount-G	Southern Biotech, Birmingham, AL, USA	Cat # 0100-01
Spectral-DAPI	Akoya biosciences, MA	
Opal 520	Akoya biosciences, MA	
Opal 540	Akoya biosciences, MA	
Opal 570	Akoya biosciences, MA	
Opal 620	Akoya biosciences, MA	
Opal 650	Akoya biosciences, MA	
Opal 690	Akoya biosciences, MA	

Table I Major resources used

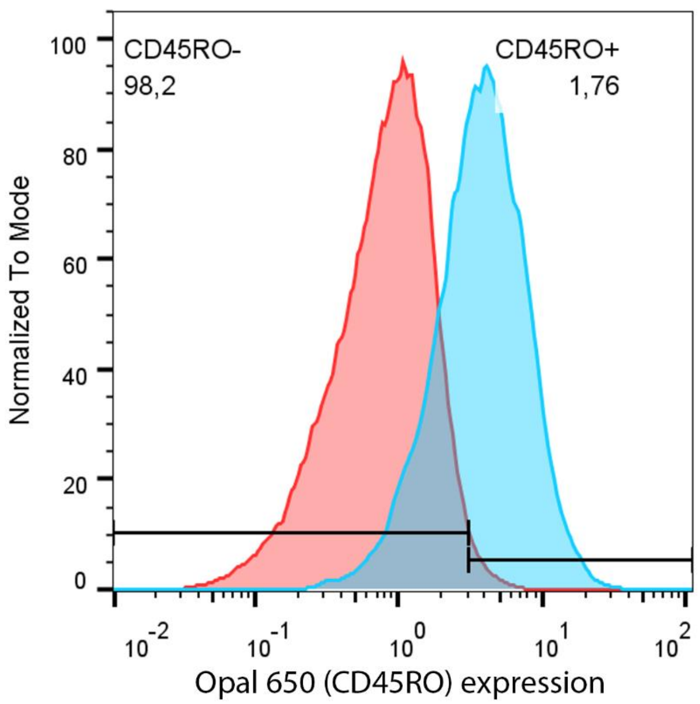

	Sample Name	Subset Name	Count
(blue)	2.fcs	T cells	10851
(red)	2.fcs	B cells$^-$ & CD1c $^-$ & T cells$^-$	57862

Figure I Representative analysis of T cell CD45RO expression. Non-immune cell population is taken as a negative control, T cells with a higher expression compared to this negative control population are considered CD45RO+ memory T cells.

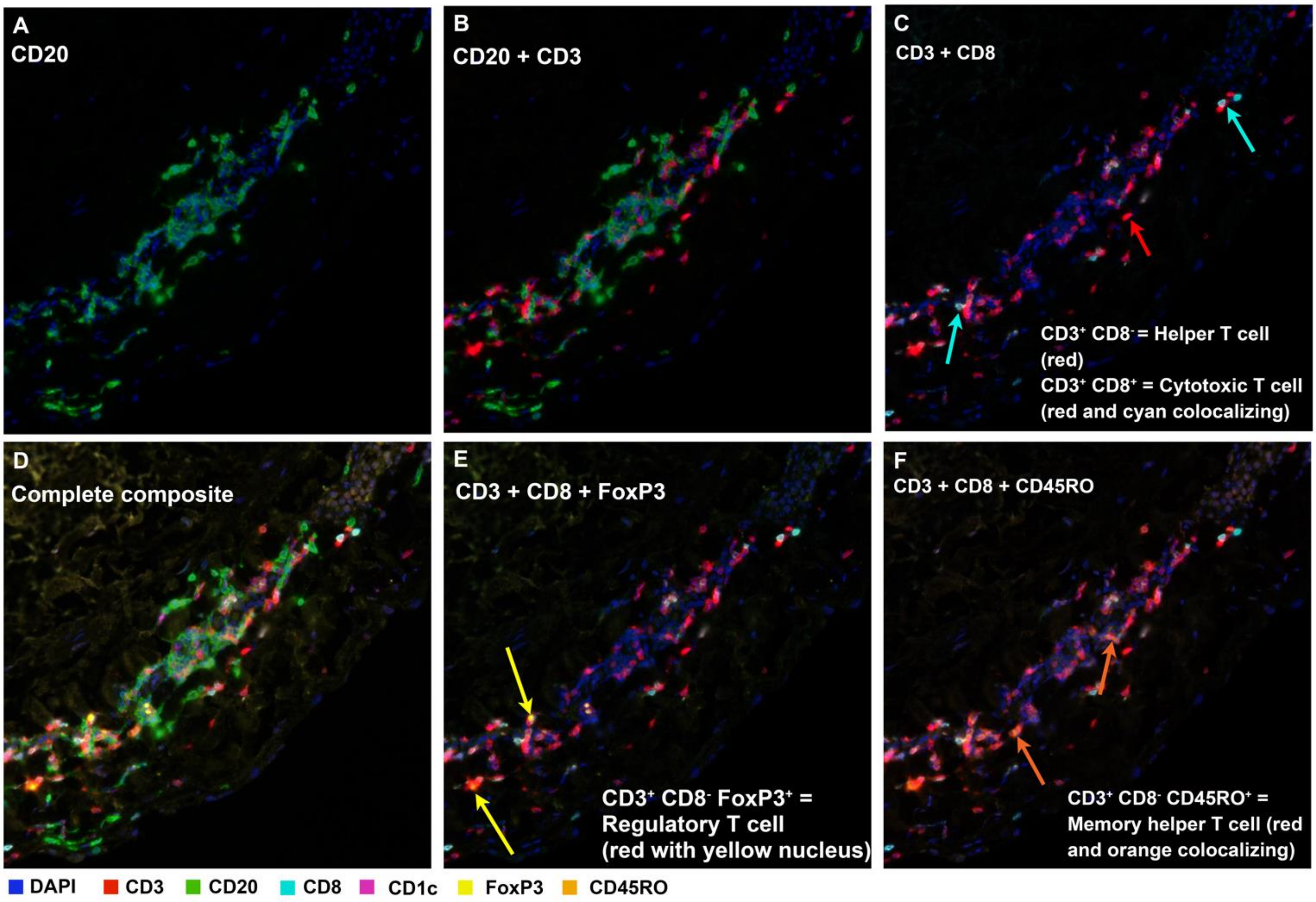

Figure II Representative 20x image displaying the various markers in false color to visualize colocalization of markers on single cells. **A** CD20 and DAPI displayed. **B** CD20, CD3 and DAPI displayed, showing no colocalization of the B cell and T cell markers. **C** CD3, CD8 and DAPI displayed, showing single positive CD3+ helper T cells (red) and double positive CD3+ CD8+ cytotoxic T cells (red and cyan colocalizing). **D** Complete composite image with all makers displayed. **E** CD3, CD8, FoxP3 and DAPI displayed showing CD3+ FoxP3+ double positive regulatory T cells (red and yellow). Note the transcription factor FoxP3 (yellow) localized in the nucleus. **F** CD3, CD8, CD45RO and DAPI displayed showing CD4+ CD8- CD45RO+ memory helper T cells (red and orange).

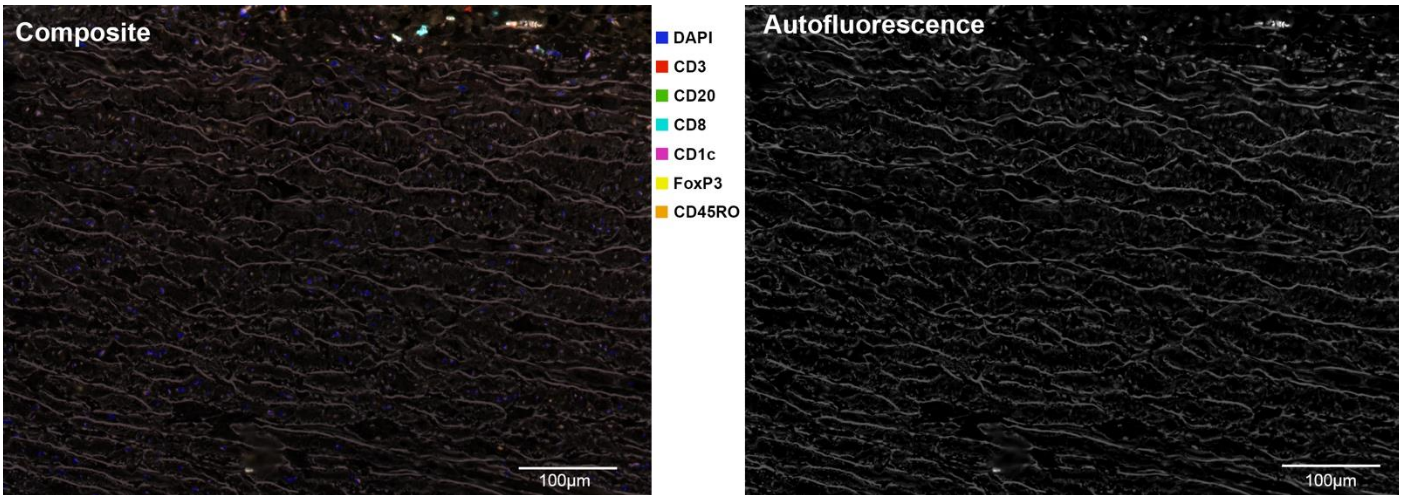

Figure III Unmixing of the autofluorescent elastin fibers (white)

A

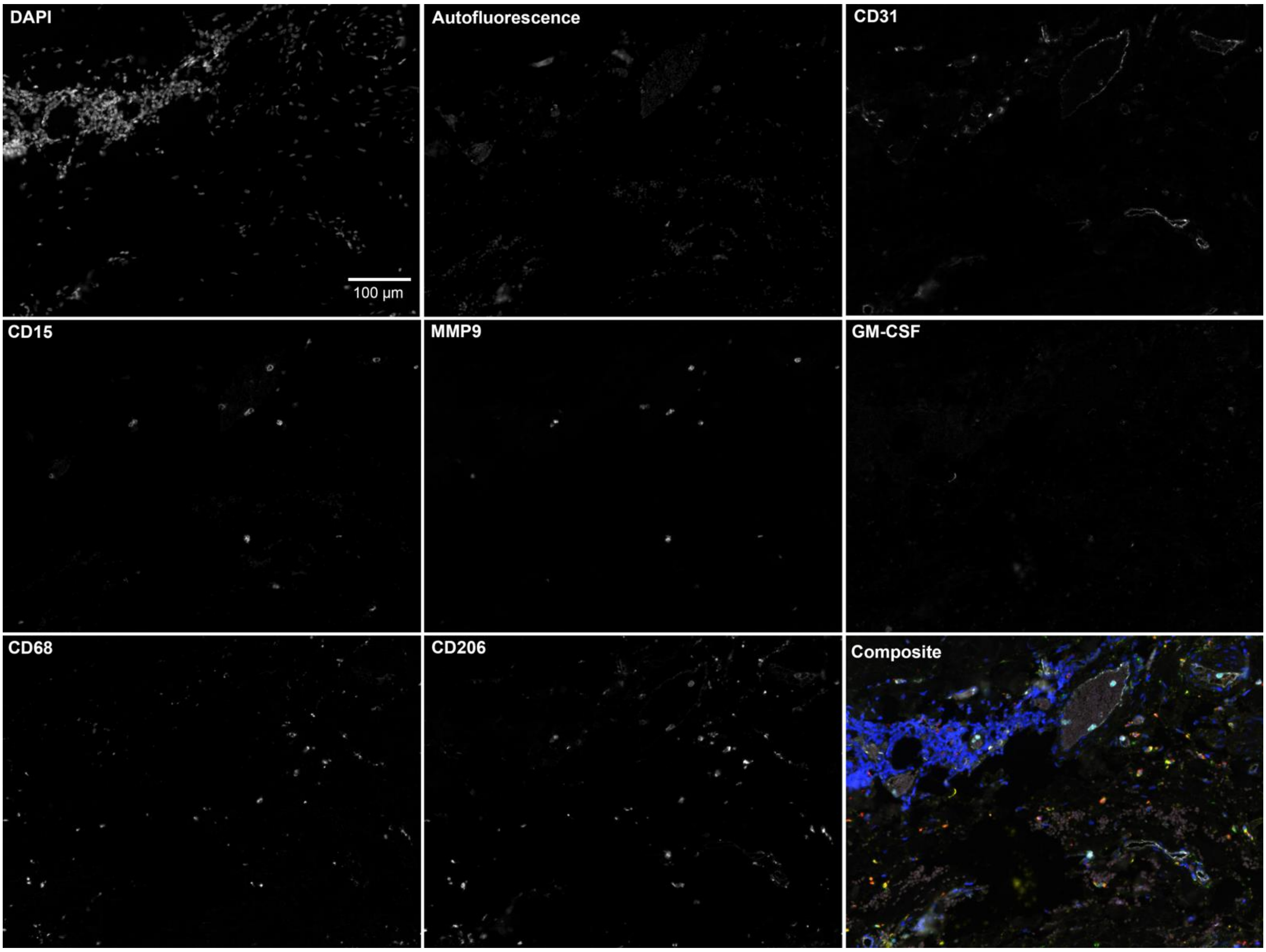
DAPI
100 µm
Autofluorescence
CD31
CD15
MMP9
GM-CSF
CD68
CD206
Composite

B

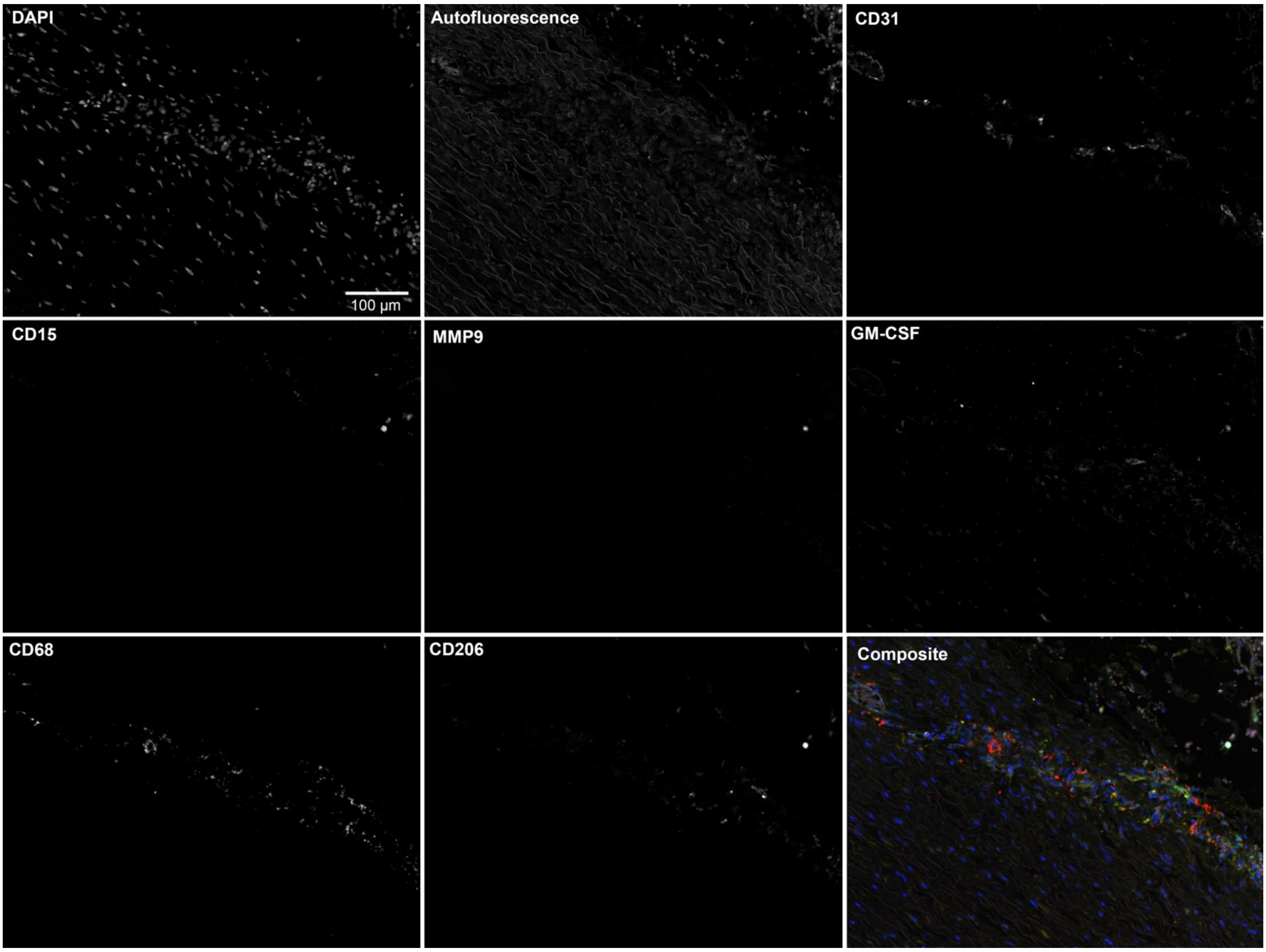
DAPI
Autofluorescence
CD31
100 µm
CD15
MMP9
GM-CSF
CD68
CD206
Composite

C

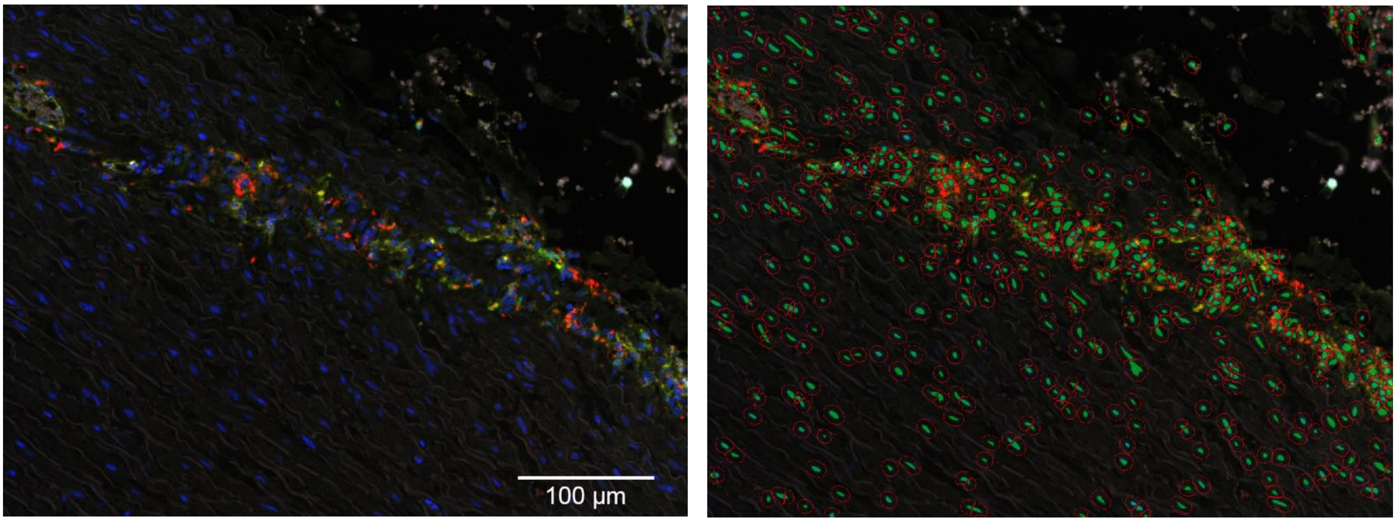
100 µm

D

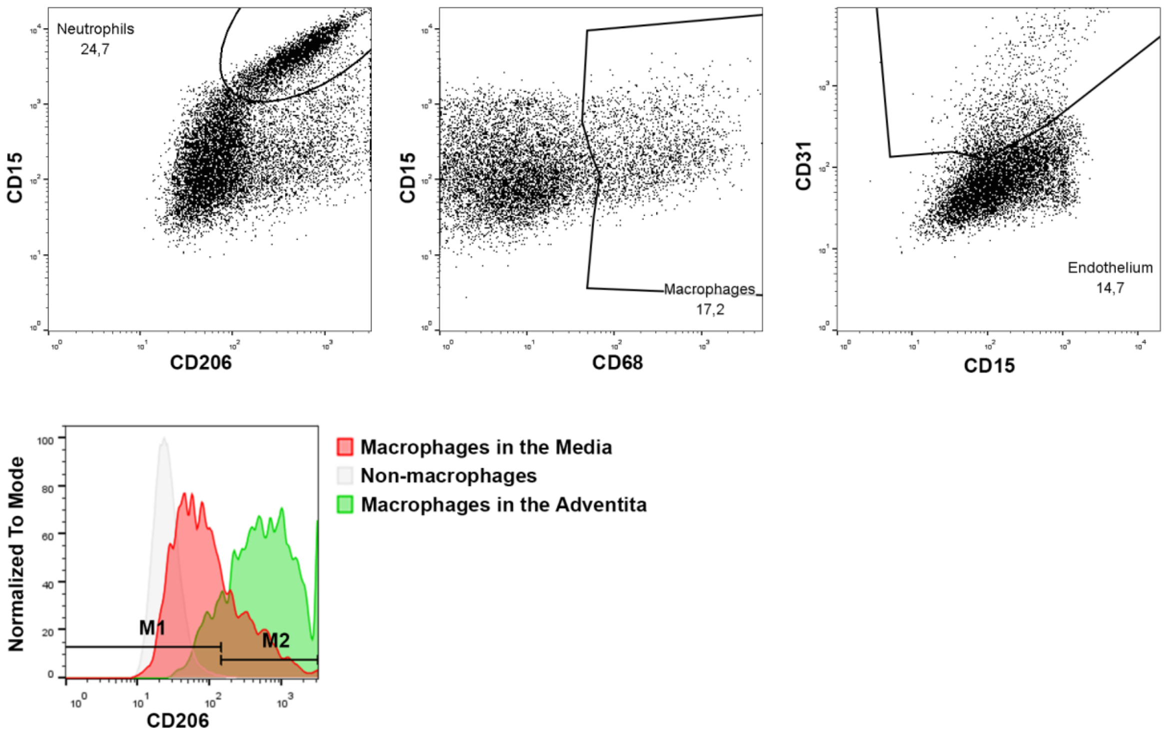

Figure IV Image processing for the innate immune system panel. **A** Representative unmixing example form the adventitia of an aneurysm sample. **B** Representative unmixing example from the adventitia of a dissection sample. **C** Cell segmentation for the innate panel. Note the larger membrane search distance compared to the adaptive immune system panel, which was chosen because of the irregular shape of macrophages. Furthermore, a cell is only recognized when a DAPI signal is present, some CD68 and CD206 signals are not accompanied by a DAPI signal because cells can span multiple 4µm slices. **D** Marker expression of the segmented single cells was assessed with a flowcytometry-like method. This resulted in cell populations that could be reproducibly gated as shown here for the innate immune cell panel. M1/M2 dichotomy gate was placed based on the non-macrophage expression of CD206 in that sample (grey).

A

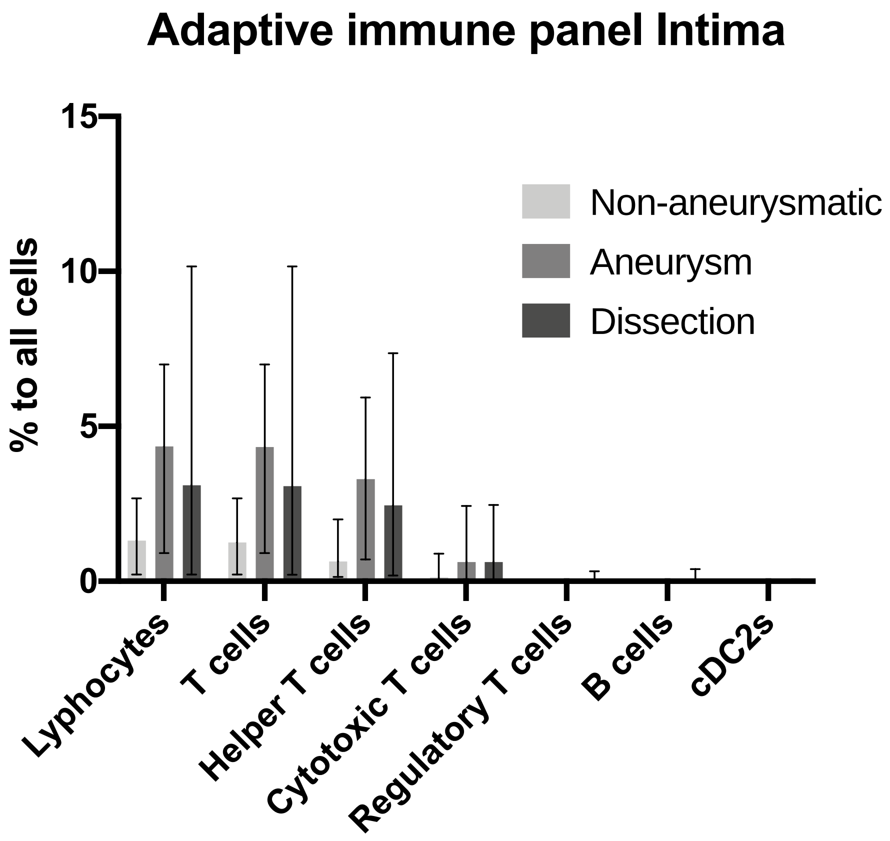

B

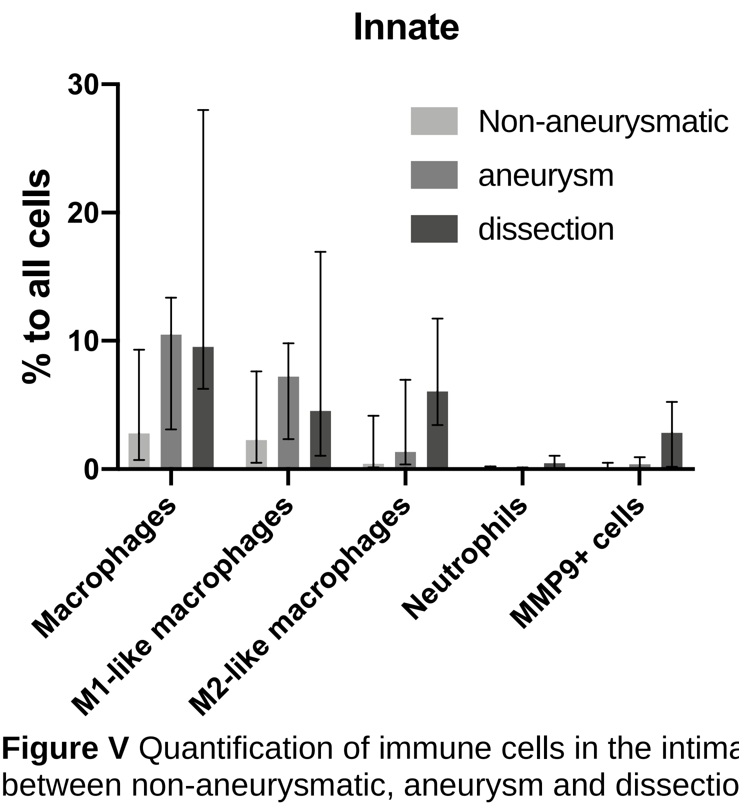

Figure V Quantification of immune cells in the intima does not show significant differences between non-aneurysmatic, aneurysm and dissection groups. **A** Quantification of the adaptive immune cells. **B** Quantification of the innate immune cells. Data as median with interquartile range.

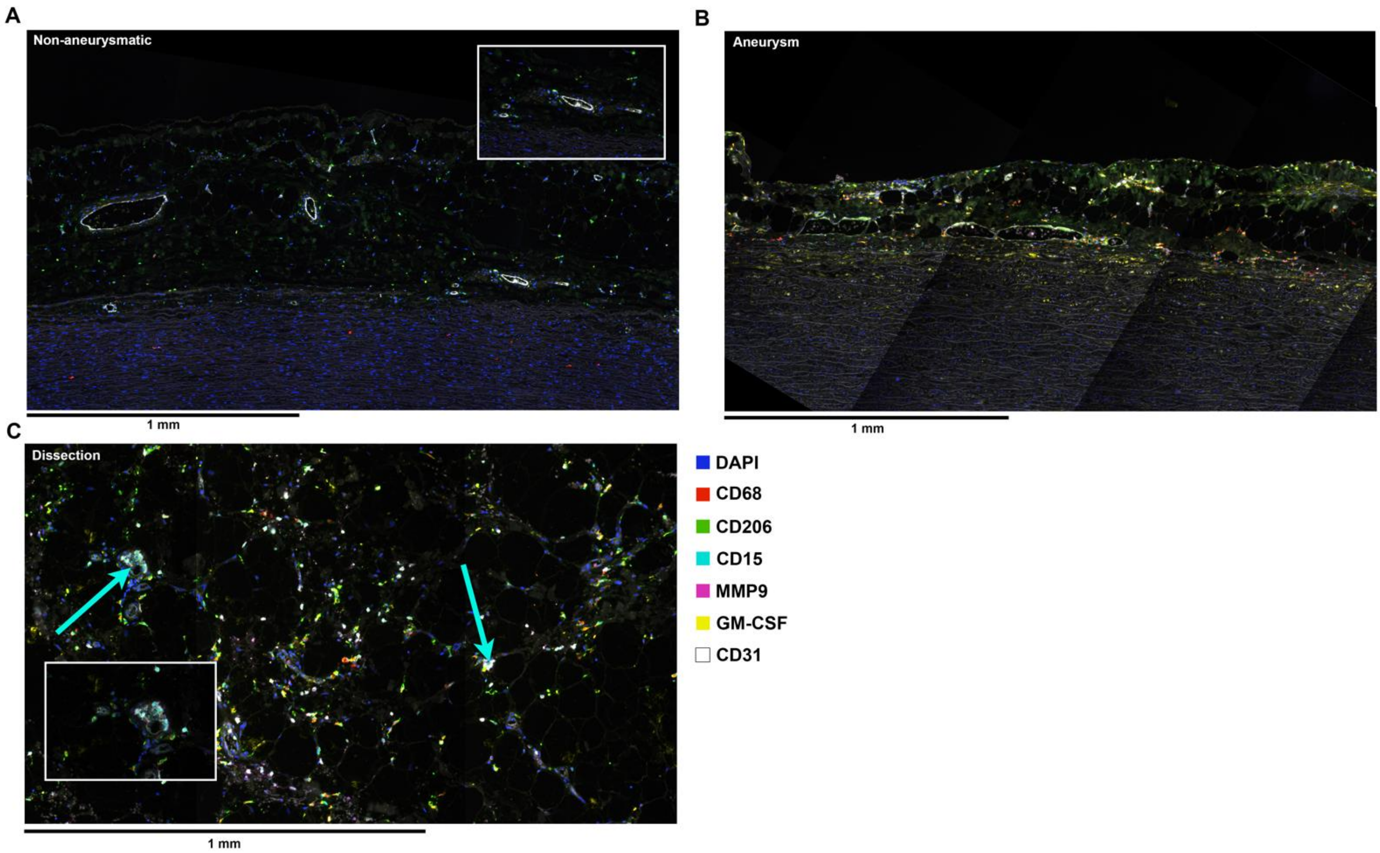

Figure VI Representative images of the adventitia from samples stained with the innate immune system panel. Only dissections show an increased number of CD15+ neutrophils and possible signs of neutrophil extravasation from the CD31+ *vaso vasorum*. **A** Adventitia of a non-aneurysmatic aorta sample of a patient with BAV. Note the sparse CD15+ (cyan) neutrophils in the CD31+ *vaso vasorum*. **B** Adventitia of an aneurysmatic aorta sample of a patient with BAV. Note an increase in CD15+ (cyan) neutrophils still present inside the CD31+ *vaso vasorum*. Furthermore, an increase in CD68+ (red) CD206+ (green) macrophages and increased expression of GM-CSF (yellow) in the media/adventitia border can be observed. **C** Adventitia of a dissected aorta from a patient with BAV. Note the steep increase in CD15+ (cyan) neutrophils that are now clearly extravasated. Signs consistent with neutrophil extravasation can be observed (cyan arrows).

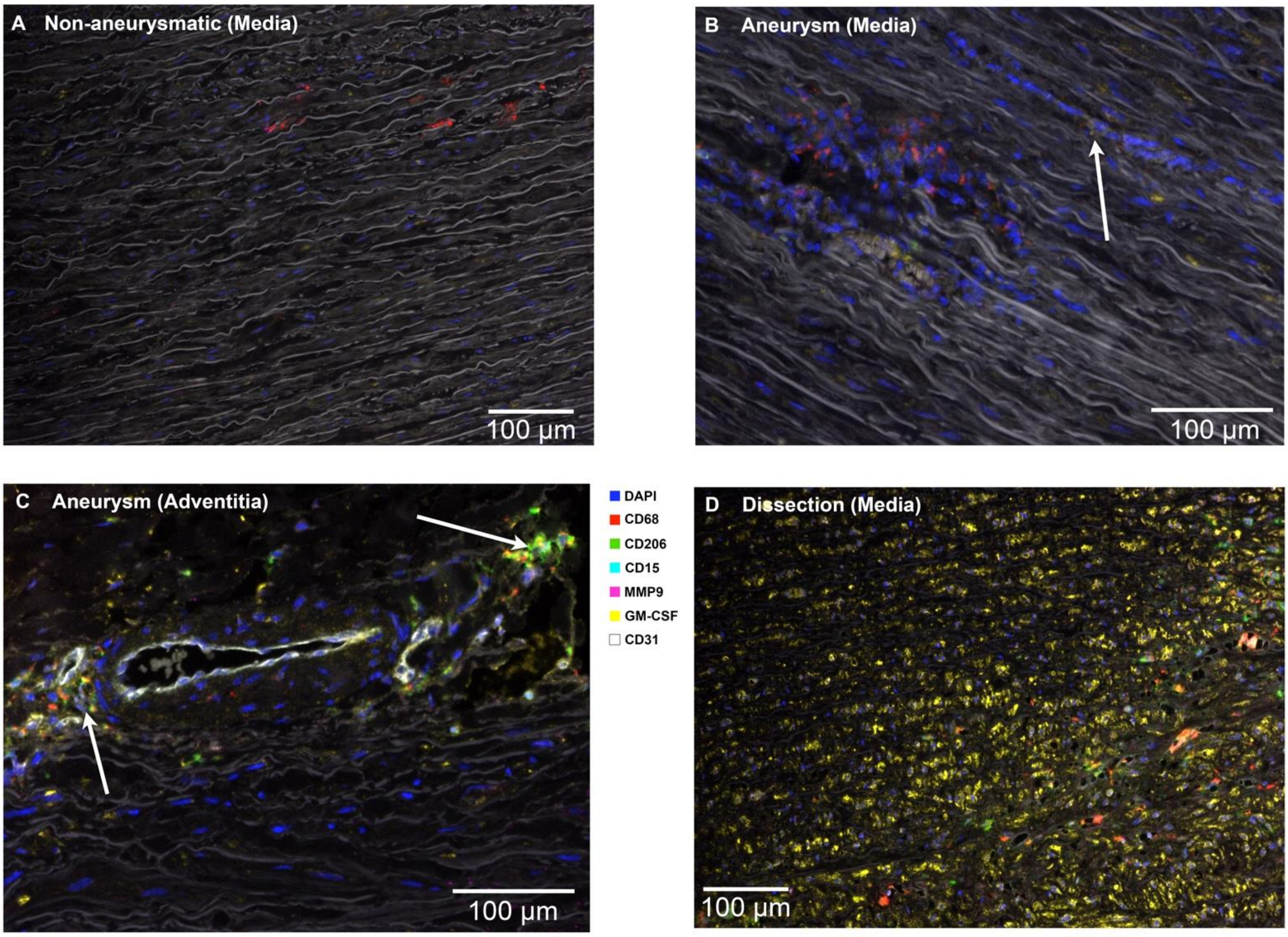

Figure VII GM-CSF expression (yellow) in non-aneurysmatic, aneurysm and dissection samples. **A** Non-aneurysmatic samples showed sporadic GM-CSF expression. **B** Aneurysm sample at the media layer with M1-like macrophage (red) accumulation near disrupted elastin fibers, some slight GM-CSF expression is visible in surrounding cells (arrow). **C** Aneurysm sample at the media-adventitia border with M1-like macrophage (red) and M2-like macrophage (red and green colocalization) accumulation in close proximity to the vasa vasorum. These macrophages show strong expression of GM-CSF (yellow) (arrows). **D** Dissection sample at the media with M1-like macrophages (red) with strong GM-CSF (yellow) expression. Strong GM-CSF expression (yellow) can be seen in most cells of the media, these cells are most likely vascular smooth muscle cells.
